# The Effect of Intensive Implementation Support on Fidelity for Four Evidence-Based Psychosis Treatments: A Cluster Randomized Trial

**DOI:** 10.1007/s10488-021-01136-4

**Published:** 2021-04-19

**Authors:** Torleif Ruud, Robert E. Drake, Jūratė Šaltytė Benth, Karin Drivenes, Miriam Hartveit, Kristin Heiervang, Tordis S. Høifødt, Vegard Ø. Haaland, Inge Joa, Jan Olav Johannessen, Karl Johan Johansen, Bjørn Stensrud, Espen Woldsengen Haugom, Hanne Clausen, Eva Biringer, Gary R. Bond

**Affiliations:** 1 Division of Mental Health Services, Akershus University Hospital, Lørenskog, Norway; 2 Institute of Clinical Medicine, Campus Ahus, University of Oslo, Oslo, Norway; 3 Westat, Lebanon, NH USA; 4 Health Services Research Unit, Akershus University Hospital, Lørenskog, Norway; 5 Division of Mental Health, Sørlandet Hospital, Kristiansand, Norway; 6 Hospital Pharmacies Enterprise, South Eastern Norway, Oslo, Norway; 7 Section of Research and Innovation, Helse Fonna Health Trust, Haugesund, Norway; 8 Department of Global Public Health and Primary Care, University of Bergen, Bergen, Norway; 9 Centre for Medical Ethics, Institute for Health and Society, University of Oslo, Oslo, Norway; 10 University Hospital of Northern Norway, Tromsø, Norway; 11 The Artic University of Norway UiT, Tromsø, Norway; 12 Clinical Neuroscience Research Group, Department of Psychology, University of Oslo, Oslo, Norway; 13 TIPS Centre for Clinical Research in Psychosis, Psychiatric Division, Stavanger University Hospital, Stavanger, Norway; 14 Faculty of Health, Network for Medical Sciences, University of Stavanger, Stavanger, Norway; 15 Mental Health Norway, Oslo, Norway; 16 Division of Mental Health, Innlandet Hospital Trust, Brumunddal, Norway; 17 Norwegian Center for Addiction Research, Institute of Clinical Medicine, University of Oslo, Oslo, Norway

**Keywords:** Psychoses, Mental health services, Evidence-based practice, Implementation support, Fidelity scale

## Abstract

**Purpose:**

Service providers need effective strategies to implement evidence-based practices (EBPs) with high fidelity. This study aimed to evaluate an intensive implementation support strategy to increase fidelity to EBP standards in treatment of patients with psychosis.

**Methods:**

The study used a cluster randomized design with pairwise assignment of practices within each of 39 Norwegian mental health clinics. Each site chose two of four practices for implementation: physical health care, antipsychotic medication management, family psychoeducation, illness management and recovery. One practice was assigned to the experimental condition (toolkits, clinical training, implementation facilitation, data-based feedback) and the other to the control condition (manual only). The outcome measure was fidelity to the EBP, measured at baseline and after 6, 12, and 18 months, analyzed using linear mixed models and effect sizes.

**Results:**

The increase in fidelity scores (within a range 1–5) from baseline to 18 months was significantly greater for experimental sites than for control sites for the combined four practices, with mean difference in change of 0.86 with 95% CI (0.21; 1.50), p = 0.009). Effect sizes for increase in group difference of mean fidelity scores were 2.24 for illness management and recovery, 0.68 for physical health care, 0.71 for antipsychotic medication management, and 0.27 for family psychoeducation. Most improvements occurred during the first 12 months.

**Conclusions:**

Intensive implementation strategies (toolkits, clinical training, implementation facilitation, data-based feedback) over 12 months can facilitate the implementation of EBPs for psychosis treatment. The approach may be more effective for some practices than for others.

**Supplementary Information:**

The online version contains supplementary material available at 10.1007/s10488-021-01136-4.

## Introduction

Evidence-based practices (EBPs) can improve treatment outcomes for patients with psychosis. However, services must adhere to EBP model principles, which is rare in daily clinical work (Bighelli et al., 2016; Weinmann et al., 2007). Researchers and policy experts have therefore proposed using fidelity scales to assess whether a practice is implemented according to the core principles and procedures defining the EBP. Although the crucial outcome of EBPs is to improve patients’ health and quality of life, fidelity is a measurable, intermediate outcome of the implementation of EBPs (Proctor et al., 2011). Fidelity scales can guide implementation and assess quality (Bond & Drake, 2020), though few studies have measured fidelity for multiple EBPs over several points in time (McHugo et al., 2007).

Routine mental health service providers typically implement EBPs with variable quality because they lack implementation supports. Clinical researchers have therefore developed theories, models, and frameworks for implementation strategies (Damschroder et al., 2009; Nilsen, 2015; Proctor et al., 2009), including strategies for evidence-based psychosocial interventions for people with severe mental illness (Menear & Briand, 2014). Strategies generally entail engaging managers and clinicians, helping practitioners to understand the needs for change, providing toolkits with a practice manual, conducting workshops to build enthusiasm and train practitioners, and offering longitudinal supervision and small group discussions based on feedback from fidelity assessments and other measurements. Experts recommend that implementation supports should be reasonably intensive, sensitive to context-specific conditions, and adjusted to the implementation phase (Menear & Briand, 2014). A compilation of Expert Recommendations for Implementation Change lists 73 implementation strategies with definitions (Powell et al., 2015), but many of these strategies are rarely used (Perry et al., 2019). The US National Evidence-Based Practices Project, using a comprehensive but small set of implementation strategies, achieved a large increase in mean fidelity for five EBPs for severe mental illness across 53 sites (McHugo et al., 2007). Implementation strategies should reflect the aims and needs of the specific project, and strategies should be reported in sufficient detail to facilitate replication (Kirchner et al., 2020; Proctor et al., 2013). Research on specific implementation strategies in general health care is becoming common, but mental health services, including for EBPs for patients with psychosis, also need studies (Powell et al., 2019). Implementation of EBPs in mental health services is needed to address the devastating impact of behavioral health disorders in the global community, and specific implementation strategies are needed to achieve this (Dixon & Patel, 2020).

### Aims

The aim of the current cluster randomized trial was to evaluate the effectiveness of intensive support to implement EBPs for the treatment of patients with psychosis in routine public mental health services. We hypothesized that experimental sites receiving intensive implementation support would achieve higher fidelity than control sites receiving usual support.

## Methods

### Study Design and Sites

We used a cluster randomized trial to examine the effect of intensive implementation support for 18 months to mental health clinical units implementing EBPs for treatment of people with psychosis (ClinicalTrials NCT03271242, registered 5 September 2017 after recruitment of the clinical units, but before completion of data collection and data analysis). Each clinical unit chose two of four core EBPs for implementation. Based on a pairwise randomization design, each site implemented one practice assigned to the experimental condition and the other practice assigned to the control condition.

Mental health clinics in six of the 19 Norwegian health trusts, serving 38% of the country’s population in urban and rural areas, participated in the study. The primary unit of analysis was 39 clinical sites providing services to adults or adolescents with psychosis (26 community mental health centers with outpatient clinics, mobile teams, and local inpatient wards; ten inpatient departments for adults with psychosis; three departments for adolescents).

The manager of each clinical unit signed a written consent to participate in the study, including consent to randomization. The Regional Committee for Medical and Health Research Ethics in Southeastern Norway (Reg. No. REK 2015/2169) and the data protection officer for each health trust approved the study, which followed the principles in the Declaration of Helsinki.

### Power Analysis

In the US National Evidence-Based Practice Project, the mean EBP fidelity increased from 2.28 (SD 0.95) at baseline to 3.76 (SD 0.78) at 12 months (personal communication from Gary Bond, Dartmouth Psychiatric Research Center, 2014). We assumed a similar mean increase in fidelity over 18 months for the experimental practices and no increase for control practices. Based on a two-tailed significance level of 5% and 90% power, we estimated that the overall hypothesis would be adequately powered with a minimum of eight sites in each arm for each practice. With 39 units as experimental sites for one practice and control sites for another, the study had sufficient power for analyses of differences for all practices combined and potentially adequate power for each of the four individual practices, assuming the average number of sites per arm for each practice was eight or nine.

### Evidence-Based Practices for Implementation

The research group selected five EBPs for patients with psychosis that met several criteria: treatment with strong evidence and/or importance in the Norwegian national guidelines on treatment for people with psychosis (Helsedirektoratet, 2013), relevance for most patients with psychosis, and already partly established or with available training programs. In May 2015, in preparation for the current study, we conducted a survey among the clinical units in the participating health trusts on their preferences regarding each of these five practices. Four of the practices were preferred by the majority of the 26 responding units. Two were medical practices (physical health care, antipsychotic medication management) that all units were already providing without measurement of quality, and two were psychosocial practices (family psychoeducation, illness management and recovery) that were new to almost all units. Thus, the four practices were previously unavailable or not implemented to evidence-based standards. We eliminated the fifth practice (individual placement and support) from inclusion in the study design because it was preferred by a minority of the clinical units. Table 1 shows a brief description or components of each of the four practices. Previous papers described the four practices in greater detail (Egeland et al., 2020; Joa et al., 2020; Ruud, 2020a, b).

**Table 1:** Characteristics of practices and components of the intervention

Components	Physical health care	Antipsychotic medication management	Family psychoeducation	Illness management and recovery
Components and characteristics of the practice	Monitoring cardiometabolic risk factors (including for diabetes, hypertension, obesity), treatment of physical illnesses, supporting physical fitness and healthy diet, supporting smoking cessation or reduction, and supporting dental and oral health	Somatic assessment, shared decision-making, choice of medication, adjusting dosage to illness phases and situations, limiting polypharmacy, monitoring effects, monitoring side effects, assessing and supporting adherence, shared list of current medication, monitoring discontinuation of medication	The patient and the family are offered psychoeducation and training in communication and problem solving together. This is done with session every other week for 6 months for single families and for 12–24 months for multifamily groups	Training program with sessions weekly or every other week for 12 months individually or in groups. Psychoeducation to improve knowledge of mental illness, relapse prevention, behavioural training to improve medication adherence, coping skills training to reduce symptoms, and social training to strengthen support
Components of the intervention for the experimental sites*				
Toolkit (ERIC: develop educational material. Distribute educational material)	A description of the components of the practice with rationale, literature references and clinical details. Key literature, presentations from the workshop, and patient information for clinical use. The toolkit was distributed to the experimental sites and was available on a website			
Clinical training and supervision (ERIC: conduct educational meetings. Provide clinical supervision)	One-day workshop by experts on the practice. Clinicians were considered to have the clinical skills, but they received updated knowledge for this practice	One-day workshop by experts on the practice. Clinicians were considered to have the clinical skills, but they received updated knowledge for this practice	Two two-day workshops by experts on the practice. Manual for family psychoeducation. Clinical supervision by telephone offered every other week for 6 months and then monthly for 6 months	Two two-day workshops by experts on the practice. Extensive manual, including worksheets for the patients. Clinical supervision by telephone offered weekly for 6 months and then every other week for 6 months
Implementation facilitation (ERIC: use advisory workgroups use an implementation advisor)	Facilitation of the implementation process and quality improvement strategies were offered by implementation facilitators as meetings on site every other week for six months and then monthly for 12 months. The facilitation model built on teaching and encouraging managers and clinicians to organize the implementation process, identify and overcome implementation barriers, plan and monitor phase specific activities using Deming’s circle and flow charts, collect data for feedback and monitoring, recognize contextual factors, tailor the implementation process, and build systems to sustain the implementation			
Feedback at baseline and after 6, 12, and 18 months (ERIC: audit and provide feedback)	A written report with fidelity scores and comments for the experimental practice was sent to the site manager within a few weeks after each 6 months fidelity assessment. Scores were discussed with the site manager to correct any misunderstandings Feedback on the results from an online questionnaire (IPAT) to clinicians on their experiences of the implementation process was sent to the site manager after every 6 months for the experimental practice if five or more of the clinicians chosen by the manager had completed the questionnaire (Hartveit et al., 2019). The feedback contained diagrams of the answers on each question and comments to help the manager understand the staff’s experience and how the manager could support the implementation process in the site			
Component available for the control sites				
Written description of the practice	A written description of all the four practice (one part of the toolkits) was sent to all clinical unit as information before they chose which two practices they would implement			

*ERIC: implementation strategies formulated and defined in Expert Recommendations for Implementing Change (Powell et al., 2015)

### Randomization

**Fig. 1:**
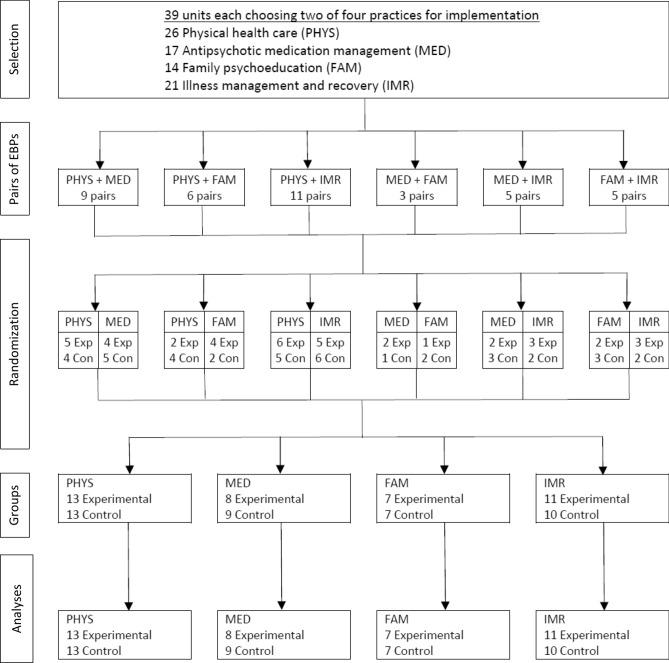
Flow diagram showing pairwise cluster-randomization of practices and units to experimental sites (Exp) and control sites (Con)

We assumed that choice would enhance motivation, following advice from the Medical Research Council in UK for local adoption of complex interventions (Craig et al., 2008). In March 2016 all 39 clinical units received a detailed description of each of the four practices to choose the two practices they wanted to implement, accepting that the unit would be randomized to experimental site for one practice and control site for the other. As shown at the top of Fig. 1, 26 units chose physical health care, 17 chose antipsychotic medication management, 14 chose family psychoeducation, and 21 chose illness management and recovery. For each clinical unit, we randomly assigned one of the chosen practices to the experimental condition (intensive implementation support) and the other to the control condition (minimal support). Thus, each clinical unit became an experimental site for one practice and a control site for the other practice. Stratified randomization achieved a balance between arms for each of the six possible pairs of two practices. Figure 1 shows a flow diagram of the randomization. Two research methodologists, blind to the identity of the 39 clinical units, conducted the randomization in April 2016. The four EBPs formed six pairs of EBPs (six different combinations of four EBPs chosen pairwise). We grouped all sites within each EBP pair and randomized them as a block to balance the number of sites assigned to each condition across blocks. We offered all sites the implementation support as planned and completed fidelity scores for all sites at four time points. We did not attempt to blind fidelity assessments.

### Intervention

As shown in Table 1, the intensive implementation support included four components: a toolkit for the practice, training for clinicians in the practice, implementation facilitation, and feedback from the fidelity assessments and from a questionnaire to clinicians on their experiences of the implementation process (Hartveit et al., 2019). The intervention period covered 18 months, from 1 September 2016 to 28 February 2018.

We distributed the printed toolkit at the start of the study to experimental sites. Experimental and control sites could access the toolkit on a website. The clinical training occurred during the first weeks of the intervention period. On average, nine to ten managers and clinicians from each site participated in the clinical workshops for their experimental practices. The average was four for family psychoeducation because a smaller number of clinicians provided the intervention. For the two psychosocial practices, trainers provided telephone supervision for 12 months after the clinical training.

Implementation facilitators visited each site every other week for 6 months and then monthly for 12 months. Each health trust recruited one to four part-time implementation facilitators to give implementation support to their participating clinical units. The facilitators were mostly mental health nurses with clinical experience working with patients with psychosis, and experience with quality improvement, but they were not experts in any of the four EBPs. In two workshops preceding the start of the intervention period, an implementation expert trained the facilitators in implementation facilitation. During the 18 months of implementation, after an initial phase with lectures and exercises, the facilitators met with the implementation expert every 6–8 weeks for further training, discussion, and networking. The implementation facilitation followed the Consolidated Framework for Implementation Research, focusing on elements and stages in the implementation process, as described in Table 1 (Damschroder et al., 2009; Grol et al., 2013; Rafferty et al., 2012). The implementation facilitators’ role was to help the sites to use quality improvement procedures in the implementation of the EBP, like it had been done in a large Dutch project on implementation of six EBPs for treatment of patients with psychosis (Harvey & Lynch, 2017; Van Duin et al., 2013).

Site leaders received feedback every 6 months for the experimental practice on fidelity and from an online questionnaire to clinicians on their experiences of the implementation process (Implementation Process Assessment Tool—IPAT) (Hartveit et al., 2019). The site leaders received no feedback for the control practice.

### Outcome Measures

The primary and only outcome measure was EBP fidelity, measured using fidelity scales for each of the four practices. Other researchers developed the Family Psychoeducation Fidelity Scale and the Illness Management and Recovery Fidelity Scale, and we reported psychometric properties for the scales elsewhere (Egeland et al., 2020; Joa et al., 2020). The current study investigators developed the Physical Health Care Fidelity Scale and the Antipsychotic Medication Management Fidelity Scale, reporting descriptions of the scales and their psychometric properties in earlier papers (Ruud, 2020a, b). The psychometrics of the four fidelity scales were good to excellent. All four fidelity scales followed the same format and scoring (Bond & Drake, 2020). Using multiple items with each rated on a 5-point behaviourally anchored continuum, a rating of 5 indicated full adherence to practice guidelines, a rating of 1 represented substantial lack of model adherence, and ratings of 4, 3, and 2 represented gradations between these two extremes. We calculated total scale scores as the unweighted sum of item scores, divided by 5. By convention, a score of 4.0 or higher is considered adequate fidelity (McHugo et al., 2007).

### Procedures

Baseline fidelity assessment occurred in May–June 2016 after the randomization and before the start of the implementation intervention in September 2016. Subsequent fidelity assessments occurred at 6, 12, and 18 months, during March–April 2017, September–October 2017, and March–April 2018. Two trained assessors rated fidelity for the two practices being implemented in each clinical unit. Fidelity assessors conducted site visits in person, rated fidelity independently, and resolved discrepancies by consensus. The fidelity visits for family psychoeducation and illness management and recovery included interviews with managers and clinicians and inspection of written material. Fidelity visits for physical health care and antipsychotic medication management included interviews with managers and clinicians and inspection of written material, using subscales to rate documentation found in 10 randomly selected patient records.

### Analyses

We described fidelity scores reporting means, confidence intervals, and distributions across all sites at baseline (before the start of the intervention) and at 18 months.

We estimated linear mixed models to analyse the overall difference between experimental and control group fidelity over time. The models included fixed effects for time, modelled as second-order polynomial to account for possible non-linear effects, group, and the interaction between the two. Models included random intercepts for units as well as random slopes for time. We used an unstructured covariance at the unit level and AR(1)-type of covariance for within-unit correlations in time. A significant interaction term implied significant differences between the groups in overall trend. Post hoc analyses assessed within-group changes between two time points and between-group differences in changes. We analysed all practices together and each of the four practices separately. We conducted residual diagnostics by assessing the residuals graphically.

We reported the results of main analyses as regression coefficients (RC), standard errors (SE) and p-values and illustrated graphically; and presented post-hoc analyses as mean within-group changes and mean differences in change between the groups with the corresponding 95% confidence intervals (CI) and p-values, and effect sizes (Cohen’s d) for the mean differences for all time intervals (Cohen, 1992). We used SPSS for Windows version 26 for descriptive analyses and SAS version 9.4 for linear mixed model analyses.

## Results

Table 2 shows the mean (CI) fidelity and distribution of fidelity scores of the four practices across all sites at baseline and at 18 months. The fidelity scores across all practices at baseline were poor. Only two (3%) of the 78 practices (39 sites with two practices each) were already implemented with adequate fidelity (4.0 or above) at baseline. One was family psychoeducation (experimental site), and one was illness management and recovery (control site). At 18 months, 13 experimental sites (33%) had reached the adequate fidelity score of 4.0 or more, compared to only two control sites (5%). Ten (77%) of the 13 experimental sites that reached an adequate fidelity score, were implementing illness management and recovery.

**Table 2:** Mean fidelity and distribution of fidelity scores for each practice at baseline and after 18 months

Scores for all sites at baseline	Sites	Fidelity score	Distribution of fidelity scores for sites N (%)					
		Mean (95% CI)	1.00	1.01–1.99	2.00–2.99	3.00–3.99	4.00–4.99	5.00
Physical health care	26	2.05 (1.87; 2.22)	0 (0.0)	14 (53.8)	12 (46.2)	0 (0.0)	0 (0.0)	0 (0.0)
Antipsychotic medication management	17	2.41 (2.21; 2.61)	0 (0.0)	2 (11.8)	14 (82.3)	1 (5.9)	0 (0.0)	0 (0.0)
Family psychoeducation	14	1.66 (1.07; 2.26)	5 (35.7)	5 (35.7)	2 (14.3)	1 (7.1)	1 (7.1)	0 (0.0)
Illness management and recovery (IMR)	21	1.34 (0.91; 1.78)	17 (81.0)	1 (4.8)	2 (9.5)	0 (0.0)	0 (0.0)	1 (4,8)
All four practices	39 × 2	1.87 (1.68; 2.05)	22 (26.2)	22 (26.2)	30 (38.5)	2 (2.6)	1 (1.3)	1 (1.3)
Fidelity scores for groups of sites at 18 months								
Experimental sites								
Physical health care	13	2.87 (2.51; 3.23)	0 (0.0)	1 (7.7)	6 (46.2)	6 (46.2)	0 (0.0)	0 (0.0)
Antipsychotic medication management	8	3.19 (2.76; 3.62)	0 (0.0)	0 (0.0)	2 (25.0)	6 (75.0)	0 (0.0)	0 (0.0)
Family psychoeducation	7	3.31 (2.00; 4.61)	0 (0.0)	2 (28.6)	0 (0.0)	2 (28.6)	3 (42.9)	0 (0.0)
Illness management and recovery (IMR)	11	4.50 (3.86; 5.15)	0 (0.0)	1 (9.1)	0 (0.0)	0 (0.0)	8 (72.7)	2 (18.2)
All experimental sites	39	3.47 (3.12; 3.83)	0 (0.0)	4 (10.3)	8 (20.5)	14 (35.9)	11 (28.2)	2 (5.1)
Control sites								
Physical health care	13	2.52 (2.26; 2.79)	0 (0.0)	1 (7.7)	10 (76.9)	2 (15.4)	0 (0.0)	0 (0.0)
Antipsychotic medication management	9	3.21 (2.99; 3.42)	0 (0.0)	0 (0.0)	1 (11.1)	8 (88.9)	0 (0.0)	0 (0.0)
Family psychoeducation	7	1.85 (0.91; 2.78)	1 (14.3)	4 (57.1)	1 (14.3)	1 (14.3)	0 (0.0)	0 (0.0)
Illness management and recovery (IMR)	10	2.16 (1.03; 3.29)	5 (50.0)	2 (20.0)	0 (0.0)	1 (10.0)	2 (20.0)	0 (0.0)
All control sites	39	2.47 (2.13; 2.80)	6 (15.4)	7 (17.9)	12 (30.8)	12 (30.8)	2 (5.1)	0 (0.0)

**Fig. 2:**
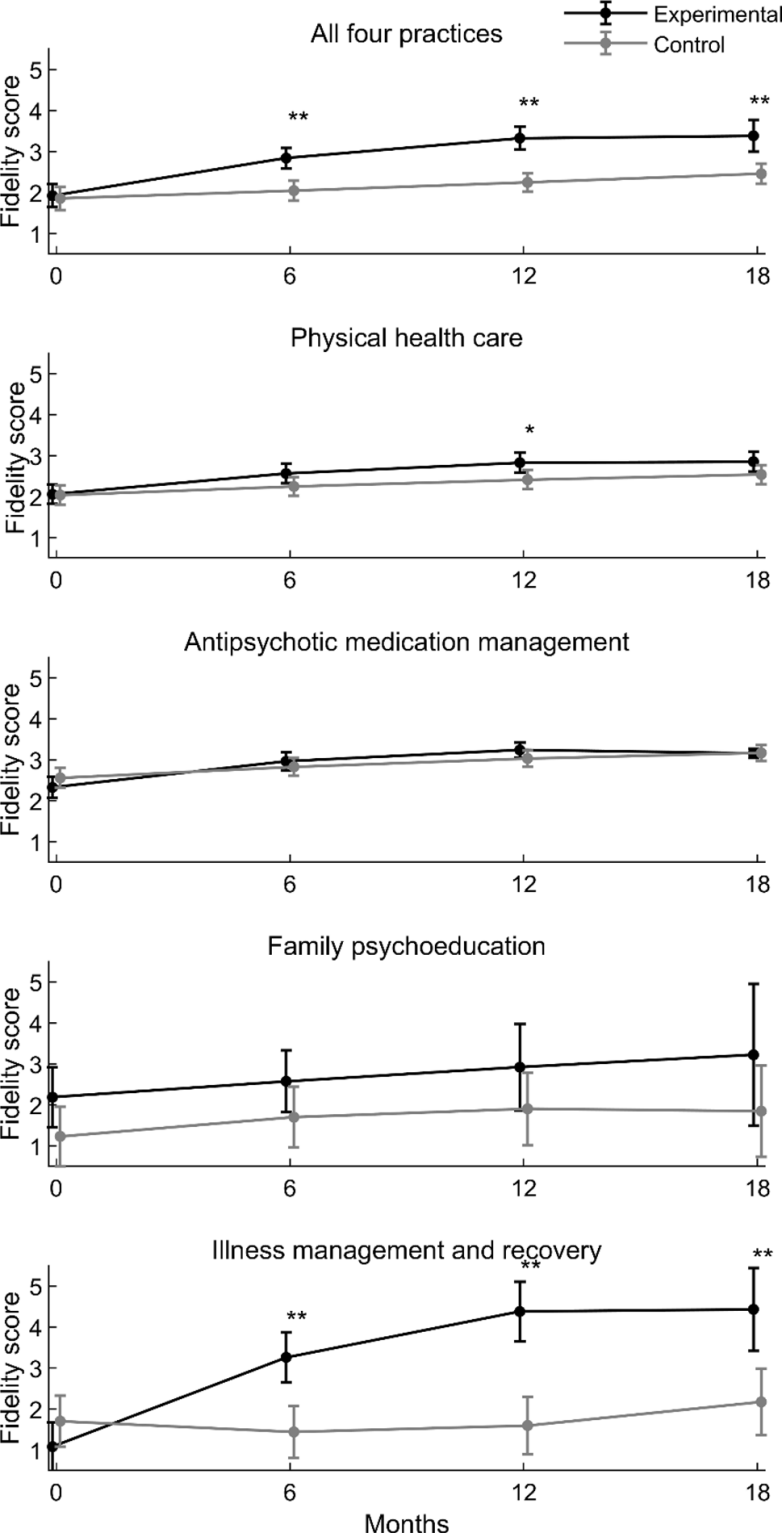
Changes and differences in fidelity scores between experimental sites and control sites from baseline to 18 months: mean, 95% CI and significance of difference at each time point (*p < 0.05, **p < 0.01)

Table 3 shows the main results of the linear mixed models assessing the difference in fidelity over time between experimental and control groups, adjusted for cluster effect on unit level. The two last rows in the table show the results for the interaction between time and groups. Large values of intraclass correlation coefficient at the unit level reflected large variation among sites for all practices. Combining the four practices, the overall increase in fidelity scores over time was significantly greater for experimental sites than for control sites. Illness management and recovery, physical health care and antipsychotic management also showed significantly greater increase in fidelity over time, while family psychoeducation did not. The greatest increase was for illness management and recovery. Figure 2 displays the differences and shows that the significant changes occurred mostly during the first 12 months.

**Table 3:** Results of linear mixed model assessing the difference of fidelity scores between intervention and control groups in time trend

Variable	All four practices		Physical health care		Antipsychotic medication management		Family psychoeducation		Illness management and recovery	
	RC (SE)	p-value	RC (SE)	p-value	RC (SE)	p-value	RC (SE)	p-value	RC (SE)	p-value
Intercept	1.85 (0.14)	< 0.001	2.04 (0.12)	< 0.001	2.56 (0.12)	< 0.001	1.23 (0.37)	0.006	1.70 (0.32)	< 0.001
Time	0.03 (0.03)	0.284	0.04 (0.02)	0.068	0.05 (0.02)	0.048	0.10 (0.09)	0.288	− 0.08 (0.06)	0.217
Time*Time	0.0002 (0.001)	0.914	− 0.0006 (0.001)	0.565	− 0.001 (0.001)	0.465	− 0.004 (0.005)	0.446	0.006 (0.003)	0.080
Group^a^	0.07 (0.22)	0.752	0.02 (0.17)	0.912	− 0.23 (0.18)	0.204	0.96 (0.52)	0.080	− 0.62 (0.44)	0.163
Time*Group	0.16 (0.05)	0.001	0.07 (0.03)	0.025	0.09 (0.03)	0.018	− 0.03 (0.13)	0.806	0.53 (0.09)	< 0.001
Time*Time*Group	− 0.006 (0.002)	0.011	− 0.003 (0.001)	0.067	− 0.004 (0.002)	0.034	0.003 (0.007)	0.652	− 0.02 (0.004)	< 0.001

^a^Control group is reference group

Table 4 shows the post hoc analyses of the changes in mean fidelity for all time intervals for the experimental and control groups and for the difference in change between the two groups. For the combined four practices the difference between experimental and control sites in mean increase in fidelity score (within a range 1–5) over 18 months was 0.86 with 95% CI (0.21–1.50), p = 0.009, with corresponding effect size 0.89 (95% CI 0.43–1.35). For illness management and recovery, the difference was 2.88 (1.89–3.87), p < 0.001, with corresponding effect size 2.24 (1.05–3.44). For physical health care the difference was 0.30 (− 0.04–0.63), p = 0.080, with corresponding effect size 0.68 (− 0.09–1.46). For antipsychotic medication management, the difference was 0.22 (− 0.12–0.57), p = 0.209, with corresponding effect size 0.71 (− 0.37–1.70). As Table 4 shows, the two later medical practices had a significant difference in increase with medium to large effect sizes during the first 12 months. For family psychoeducation, we detected no significant changes over time and only small effect sizes. None of the practices showed a significant difference in change from 12 to 18 months. Figure 2 illustrates the changes reported in Table 4.

**Table 4:** Post hoc analyses of fidelity changes over time within groups and between groups

Time interval	Experimental group		Control group		Experimental group vs. Control group		
	Mean change (95% CI)	p-value	Mean change (95% CI)	p-value	Mean diff. in change (95% CI)	p-value	Effect size (95% CI)
All four practices							
0–6 months	0.92 (0.68; 1.16)	< 0.001	0.19 (− 0.04; 0.42)	0.113	0.73 (0.34; 1.12)	< 0.001	0.87 (0.41; 1.32)
0–12 months	1.40 (1.07; 1.74)	< 0.001	0.39 (0.09; 0.69)	0.010	1.01 (0.48; 1.55)	< 0.001	1.19 (0.72; 1.66)
0–18 months	1.46 (1.03; 1.89)	< 0.001	0.60 (0.28; 0.92)	< 0.001	0.86 (0.21; 1.50)	0.009	0.89 (0.43; 1.35)
6–12 months	0.49 (0.34; 0.63)	< 0.001	0.20 (0.09; 0.31)	< 0.001	0.29 (0.07; 0.50)	0.009	0.36 (− 0.09; 0.81)
6–18 months	0.54 (0.16; 0.92)	0.005	0.41 (0.10; 0.72)	0.009	0.13 (− 0.43; 0.69)	0.647	0.14 (− 0.30; 0.59)
12–18 months	0.06 (− 0.21; 0.33)	0.685	0.21 (− 0.03; 0.45)	0.085	− 0.16 (− 0.56; 0.25)	0.455	− 0.17 (− 0.61; 0.28)
Physical health care							
0–6 months	0.51 (0.33; 0.68)	< 0.001	0.21 (0.04; 0.38)	0.014	0.30 (0.05; 0.54)	0.018	0.69 (− 0.09; 1.46)
0–12 months	0.77 (0.57; 0.98)	< 0.001	0.38 (0.17; 0.58)	0.010	0.40 (0.07; 0.72)	0.016	0.90 (0.12; 1.68)
0–18 months	0.80 (0.65; 0.94)	< 0.001	0.50 (0.31; 0.69)	< 0.001	0.30 (− 0.04; 0.63)	0.080	0.68 (− 0.09; 1.46)
6–12 months	0.27 (0.22; 0.31)	< 0.001	0.17 (0.10; 0.23)	< 0.001	0.10 (− 0.01; 0.21)	0.080	0.23 (− 0.54; 1.00)
6–18 months	0.29 (0.15; 0.43)	< 0.001	0.29 (0.11; 0.47)	0.002	0.00 (− 0.28; 0.29)	0.988	0.01 (− 0.76; 0.77)
12–18 months	0.03 (− 0.11; 0.16)	0.704	0.12 (− 0.03; 0.27)	0.107	− 0.10 (− 0.32; 0.12)	0.389	− 0.22 (− 0.99; 0.55)
Antipsychotic medication management							
0–6 months	0.64 (0.44; 0.84)	< 0.001	0.27 (0.08; 0.46)	0.005	0.37 (0.08; 0.65)	0.011	1.05 (0.06; 2.04)
0–12 months	0.91 (0.70; 1.13)	< 0.001	0.47 (0.27; 0.68)	< 0.001	0.44 (0.09; 0.78)	0.013	1.34 (0.35; 2.33)
0–18 months	0.83 (0.69; 0.97)	< 0.001	0.61 (0.45; 0.77)	< 0.001	0.22 (− 0.12; 0.57)	0.209	0.71 (− 0.27; 1.70)
6–12 months	0.28 (0.23; 0.32)	< 0.001	0.20 (0.15; 0.26)	< 0.001	0.07 (− 0.04; 0.19)	0.209	0.24 (− 0.74; 1.22)
6–18 months	0.20 (− 0.02; 0.41)	0.071	0.34 (0.12; 0.56)	0.002	− 0.14 (− 0.49; 0.20)	0.412	− 0.50 (− 1.48; 0.48)
12–18 months	− 0.08 (− 0.28; 0.12)	0.419	0.14 (− 0.06; 0.33)	0.170	− 0.22 (− 0.50; 0.06)	0.130	− 0.83 (− 1.81; 0.15)
Family psychoeducation							
0–6 months	0.39 (− 0.38; 1.15)	0.319	0.47 (− 0.28; 1.22)	0.223	− 0.08 (− 1.14; 0.98)	0.882	− 0.08 (− 1.13; 0.97)
0–12 months	0.73 (− 0.40; 1.86)	0.204	0.67 (− 0.30; 1.65)	0.176	0.06 (− 1.27; 1.39)	0.930	0.05 (− 1.00; 1.10)
0–18 months	1.03 (− 0.68; 2.75)	0.237	0.61 (− 0.47; 1.70)	0.268	0.42 (− 0.96; 1.80)	0.552	0.27 (− 0.79; 1.33)
6–12 months	0.34 (− 0.23; 0.92)	0.237	0.20 (-0.16; 0.57)	0.268	0.14 (− 0.32; 0.60)	0.552	0.12 (− 0.93; 1.17)
6–18 months	0.65 (− 0.91; 2.20)	0.416	0.15 (-0.85; 1.14)	0.774	0.50 (− 0.81; 1.81)	0.455	0.32 (− 0.74; 1.38)
12–18 months	0.30 (− 0.77; 1.37)	0.583	− 0.06 (− 0.83; 0.71)	0.881	0.36 (− 0.69; 1.41)	0.500	0.22 (− 0.84; 1.27)
Illness management and recovery							
0–6 months	2.18 (1.68; 2.67)	< 0.001	− 0.26 (− 0.77; 0.25)	0.314	2.44 (1.73; 3.15)	< 0.001	2.40 (1.30; 3.51)
0–12 months	3.30 (2.61; 3.98)	< 0.001	− 0.11 (− 0.75; 0.54)	0.746	3.40 (2.49; 4.31)	< 0.001	3.10 (1.79; 4.41)
0–18 months	3.35 (2.42; 4.28)	< 0.001	0.47 (− 0.20; 1.14)	0.169	2.88 (1.89; 3.87)	< 0.001	2.24 (1.05; 3.44)
6–12 months	1.12 (0.81; 1.43)	< 0.001	0.16 (− 0.07; 0.38)	0.169	0.96 (0.63; 1.29)	< 0.001	0.87 (− 0.02; 1.75)
6–18 months	1.17 (0.33; 2.02)	0.007	0.73 (0.10; 1.37)	0.024	0.44 (− 0.48; 1.36)	0.348	0.34 (− 0.51; 1.18)
12–18 months	0.05 (− 0.55; 0.66)	0.860	0.58 (0.07; 1.08)	0.026	− 0.52 (− 1.24; 0.19)	0.153	− 0.38 (− 1.23; 0.47)

## Discussion

This study demonstrated that intensive implementation support can facilitate significantly higher fidelity than usual procedures, supporting the study hypothesis. The effect was large for one of the four practices, medium to large for two practices, and absent for one practice. The significant changes occurred mostly during the first 6–12 months of intervention, and only one third of the experimental sites reached an adequate fidelity score of 4.0 after 18 months.

The parsimonious interpretation of our results is that intensive implementation supports can improve the fidelity of EBPs for patients with psychosis. However, the effects may vary for specific EBPs, which we consider below, and which has also been found in other studies of implementation support for multiple practices (McHugo et al., 2007; Van Duin et al., 2013).

Although many studies have demonstrated increased fidelity over time for a variety of EBPs (Bond & Drake, 2020), few randomized trials have evaluated the effectiveness of a defined package of intensive implementation strategies to achieve this goal. The US National Evidence Based Practice Project previously found a strong increase in fidelity over time for five EBPs, including 55% of the sites reaching an adequate fidelity score after 24 months, but the US study lacked a control group for comparison (McHugo et al., 2007). A recent cluster randomized study on implementation support for integrated treatment of concurrent mental health and substance use disorders found a moderate effect for experimental sites compared to control sites on a waiting list (Assefa et al., 2019). A recent trial comparing the effect of three levels (combinations) of implementation support for cardiovascular treatment over 12 months in community clinics found no significant differences in effect among the three levels of implementation support, but some differences compared with non-study control clinics (Gold et al., 2019).

The current study showed marked differences in combined fidelity improvements for the four practices. Illness management and recovery had a large effect of the implementation support compared to the other practices. Several factors may have contributed to this. The intervention is straightforward, primarily using a psychoeducational model. The baseline fidelity scores were low because sites were not previously using the model. The toolkit included a detailed manual, telephone supervision was given for 12 months, and many sites wanted to learn and use the practice. The large effect for the combined practices was to a large extent due to the effect for illness management and recovery.

The implementation supports for physical health care and antipsychotic medication management showed significant medium to large effects. These two interventions are complex, requiring considerable clinical judgment and shared decision-making, and both had higher baseline fidelity scores than the psychosocial practices because the medical practitioners were already providing these services. In addition, fidelity assessments using patient records may have made it more difficult to achieve high fidelity scores due to lack of documentation rather than lack of implementation. Nevertheless, these two practices still achieved significant effects over time. We have not found a comparable study on the effect of implementation support on fidelity to an evidence-based model of physical health care. Our medium effect of implementation support on antipsychotic medication management fidelity was similar to what was found in a study using another fidelity scale for medication management in the treatment of schizophrenia (Howard et al., 2009).

The implementation support for the family psychoeducation showed a lack of significant changes and small effect sizes. The weak result may have occurred because of serious confounds: one of the seven experimental sites was already implementing the practice at baseline, two experimental sites decided not to implement the practice, and the total number of sites was small. Small numbers and poor compliance may have undermined the experiment for this practice.

The current study had several strengths: it was one of few randomized controlled trials assessing an intensive implementation support strategy for implementing EBPs for the treatment of patients with psychosis. In addition, it used random assignment to a clearly defined implementation approach supported by an extensive literature review, a representative sample of routine public mental health service units with limited additional resources, the inclusion of four core EBPs, implementation support over 18 months, and extensive efforts to measure fidelity with well validated scales.

Several limitations also warrant attention. The small sample lacked power to detect differences between groups for some practices, the EBPs may have differed in difficulty of implementation, and the fidelity scales may have been non-comparable (Egeland et al., 2020; Joa et al., 2020; Ruud, 2020a, b). In addition, two sites chose practices to implement that they were already implementing at adequate fidelity at baseline, precluding the possibility of significant improvement. Further, the design with pairwise randomization within each clinical unit may have resulted in treatment contamination within sites and influenced the implementation of the control practice. Finally, generalization from Norway, a high-income country with strong government support for mental health care, may be limited.

## Conclusions

The study showed that intensive implementation support can improve the fidelity of EBPs in routine mental health services but with variability across practices. The effect was most apparent during the first 12 months. We recommend that future studies examine different components of implementation strategies.

## Guidelines Followed

The study followed the Consort Extension guidelines for cluster randomized trials, and the completed checklist for such studies are submitted together with the manuscript.

## Supplementary Information

Below is the link to the electronic supplementary material.

Supplementary file1 (DOCX 34 kb)

## Data Availability

The data is not available for distribution to others than the research group who conducted the study. Other researchers who want access to the data may contact the principal investigator (TR), who will answer whether the requested data may be made available.
